# Iron Deficiency is Not Associated with Increased Blood Cadmium in Infants

**DOI:** 10.1186/2052-4374-26-3

**Published:** 2014-02-10

**Authors:** Jung-Hun Park, Sangkyu Park, Yangho Kim

**Affiliations:** 1 Department of Occupational and Environmental Medicine, Ulsan University Hospital, University of Ulsan College of Medicine, 290-3 Cheonha-Dong, Dong-Gu, Ulsan 682-060, South Korea; 2 Department of Pediatrics, Ulsan University Hospital, University of Ulsan College of Medicine Ulsan, South Korea

**Keywords:** Iron, Deficiency, Cadmium, Anemia

## Abstract

**Objectives:**

To determine whether blood cadmium concentration is elevated in iron-deficient infants.

**Methods:**

Blood cadmium and serum ferritin concentrations, serum iron/total iron-binding capacity (Fe/TIBC) and complete blood counts were measured in 31 iron deficient and 36 control infants, aged 6–24 months. All 31 iron-deficient infants received iron supplementation for 1–6 months.

**Results:**

Blood cadmium concentrations were measured again in 19 of the iron deficient infants after their ferritin levels returned to the normal range. The mean blood cadmium concentration did not differ significantly in iron deficient and control infants. The mean blood cadmium concentration in the 19 iron-deficient infants was not significantly altered by ferric hydroxide treatment, while their hemoglobin, ferritin, and Fe/TIBC (%) concentrations were significantly higher after than before treatment.

**Conclusion:**

These findings indicate that iron deficiency does not increase blood cadmium concentrations in infants, in contrast with the effects of iron deficiency on manganese and lead concentrations.

## Introduction

Iron deficiency affects approximately one-third of the world’s population [1], occurring most frequently in infants aged 6 months to 3 years due to their rapid growth and inadequate dietary intake of iron [2]. Iron-deficiency anemia has been associated with poor cognitive development, poor motor development and behavioral problems [3,4].

Because iron is absorbed via mechanisms similar to those of other divalent metal ions, including cadmium, manganese, and lead, a dietary deficiency in iron can lead to excess absorption of cadmium, a widespread toxicant with detrimental effects on health. Indeed, iron deficiency has been found to predispose animals to cadmium toxicity by increasing gastrointestinal cadmium absorption [5-10]. Several studies have suggested an association between iron status and blood cadmium concentration in adults, especially pre-menopausal women [11-20], whereas few studies have been conducted in children [12,21-24]. We assessed whether blood cadmium concentrations are higher in iron-deficient than in control infants, and whether treatment of the former with iron supplements affects cadmium concentrations.

## Materials and methods

### Materials

Thirty one infants with iron deficiency (serum ferritin concentration < 15 μg/L), ranging in age from 6 months to 2 years, were selected from infants being treated at an ambulatory pediatric hematology clinic at Ulsan University Hospital, Ulsan, South Korea. Thirty-six healthy, age- and sex-matched control subjects, with serum ferritin concentrations higher than 15 μg/L, were selected from among the infants visiting a general pediatric clinic in the same hospital. Subjects were excluded if they were delivered preterm or at low birth weight; had a history of any disease, or had concurrent acute or chronic infection or inflammation; or if their parents had a history of occupational exposure to cadmium. All 31 iron-deficient infants were treated with ferric hydroxide-polymaltose complex (6 mg/kg Fe^3+^/day) for 1–6 months. Blood cadmium concentrations were determined in all control subjects and in all iron-deficient subjects prior to iron supplementation. Blood cadmium concentrations were assessed again in 19 iron-deficient infants after their ferritin concentrations returned to the normal range; the other 12 iron-deficient infants were lost to follow up. Parents of infants in both groups provided written informed consent, and the study protocol was approved by the institutional review board of Ulsan University Hospital.

### Laboratory testing

A questionnaire was administered to the parents of each infant to obtain basic perinatal information, along with information on breast feeding and nutrition. Heparinized venous blood samples were obtained from each infant, and blood count, hemoglobin concentration, hematocrit, serum ferritin concentration, and serum iron/total iron-binding capacity (Fe/TIBC) were measured. Iron deficiency was defined as serum ferritin < 15 μg/L [25].

### Cadmium determination in whole blood

Blood cadmium concentration was determined by flameless graphite furnace atomic absorption spectrophotometry (AAS) (Spectra AA880-GTA 100, Varian, Australia), using the standard addition method. Briefly, aliquots (0.1 ml) of blood were diluted 20 fold with 0.1% (v/v) Triton X-100, and 15-μL samples were injected into the graphite furnace. All blood cadmium analyses were carried out in the Ulsan University Hospital laboratory, which had passed the Quality Assurance Program (for cadmium) operated by the Korea Occupational Safety and Health Agency. The detection limit for blood cadmium in the present study was 0.09 μg/L. Two samples had cadmium levels below the detection limit; in those samples, we considered the level in the sample to be the detection limit divided by the square root of 2 [26].

### Statistical analyses

Blood cadmium levels were natural log-transformed because their distributions were skewed, and the geometric means (GMs) are presented. Significant differences in the means of continuous variables between iron-deficient and control infants were determined using Student’s t-tests. Differences in the proportion of male infants between these groups were determined using the chi-square test. Significant differences in the means of variables before and after iron therapy were determined using paired t tests. SPSS (v14) software was used for all statistical analyses, and a *P* value <0.05 was considered significant.

## Results

Age and gender distribution were similar in the iron-deficient and control groups, as were their GM blood cadmium concentrations. However, hemoglobin concentrations, hematocrit levels, and serum ferritin levels differed significantly in the two groups (Table 1).

**Table 1:** Demographic, clinical and laboratory features of the study subjects

**Group characteristics**	**Iron-deficient infants (n = 31)**	**Control (n = 36)**	***P *** **value**
Age (months)	12.0 ± 3.7	11.9 ± 4.1	0.867
Male (%)	65.6	52.8	0.330
Breast feeding (months)	10.3 ± 3.4	6.3 ± 4.9	<0.001
Cadmium (μg/L)*	0.56 (0.06-1.20)	0.53 (0.06-1.84)	0.724
Hemoglobin (g/dL)	10.49 ± 1.84	11.83 ± 1.02	0.001
Hematocrit (%)	32.70 ± 4.08	35.02 ± 2.60	0.008
Ferritin (μg/L)	7.4 ± 3.0	39.1 ± 23.8	<0.001
Fe (μg/dL)	39.09 ± 29.56	47.14 ± 27.79	0.255
TIBC (μg/dL)	413.19 ± 53.18	351.80 ± 47.07	<0.001
Fe/TIBC (%)	10.2 ± 8.6	13.7 ± 8.5	0.091

Results reported as mean ± SD *geometric mean (range).

Mean duration of breast feeding was longer in iron-deficient than in control infants. All the iron-deficient infants were treated with an iron supplement, and 19 were tested again for blood cadmium concentrations after their ferritin concentrations reached the normal range. The GM blood cadmium concentration in these 19 infants was not significantly altered by ferric hydroxide treatment, while their hemoglobin, ferritin, and Fe/TIBC (%) levels were significantly higher after than before treatment (Table 2).

**Table 2:** Laboratory features of iron-deficient infants before and after iron therapy

**Group characteristics**	**Before iron therapy (n = 19)**	**After iron therapy (n = 19)**	***P *** **value**
Cadmium (μg/L) *	0.58 (0.06-1.40)	0.47 (0.06-1.38)	0.529
Hemoglobin (g/dL)	10.49 ± 1.75	12.38 ± 0.85	0.001
Hematocrit (%)	32.77 ± 3.80	36.87 ± 2.24	0.002
Ferritin (μg/L)	7.03 ± 3.16	20.83 ± 7.04	<0.001
Fe (μg/dL)	35.11 ± 25.30	64.68 ± 36.61	0.006
TIBC (μg/dL)	416.68 ± 46.50	365.89 ± 52.79	<0.001
Fe/TIBC (%)	8.8 ± 6.8	17.9 ± 9.5	0.001

Results reported as mean ± SD and compared by paired t tests. *geometric mean (range) Iron-deficient infants were followed-up after 1–6 months of treatment with ferric hydroxide-polymaltose complex (6 mg/kg Fe^3+^ per day).

## Discussion

Cadmium concentrations have been reported to increase as iron stores decrease in premenopausal women [11-20]. However, no association between iron deficiency and elevated cadmium levels was observed in menopausal women [27-29] or in men [19,30], and few studies to date have analyzed the association between iron deficiency and elevated cadmium levels in children. Furthermore the studies that have been performed in children have yielded conflicting results. Some studies reported an association [21,24] between iron deficiency and cadmium, whereas others found no such association [22,23], and one study reported an association between ferritin and cadmium concentrations in female adolescents only [12]. To our knowledge, our study is the first to assess the association between iron deficiency and cadmium concentration in infants, finding that the two were not associated. In contrast, assessment of the same study subjects showed that iron deficiency was associated with increased blood lead and manganese concentrations [31,32]. The GM of blood cadmium (0.54 μg/L) in all infants in the present study was similar to that of adults aged 20–29 years (0.56 μg/L), and lower than that of adults aged 20 years and older (0.93 μg/L) [20].

Our finding, that iron deficiency and cadmium concentration were not associated in infants, is compatible with some previous studies in children [22,23] but not with others [21,24]. These discrepancies may be due in part to differences in cadmium exposure levels or the age distribution of study subjects. For example, the two studies finding an association between iron deficiency and cadmium in children involved subjects living in an area of Turkey with heavy air pollution [21] and in children with blood cadmium concentrations more than 6-fold higher than in our study cohort [24]. The subjects assessed in the present study consisted of infants living in a non-polluted area, and with very low blood cadmium concentrations. Furthermore, the subjects in most previous studies included children and/or adolescents, but not infants [12,21-24].

The placenta may act as a partial barrier to fetal exposure to cadmium [33], and only 5-10% of maternal blood cadmium may be transferred to human milk because of metallothionein binding of cadmium in blood cells [34]. Cadmium concentrations usually increase with age [20,30,35,36]. Thus, infants may have smaller chances of exposure to cadmium, and may not show increased blood cadmium concentrations associated with iron deficiency. In contrast, lead can pass the placental barrier and is present in more widespread sources to which infants may be exposed than cadmium, resulting in lead being more easily absorbed by infants with iron deficiency [32]. Manganese, an essential element, is abundant in foods, and is easily absorbed in individuals with iron deficiency [37-40].

Our results suggest that iron deficiency is associated with prolonged breast feeding in infants, a result consistent with findings showing that prolonged breast feeding was associated with iron deficiency [41,42]. Since not all infants in the present study who received prolonged breast feeding were iron deficient, differences in the iron status of these infants may depend on whether they receive iron-rich supplementary foods during prolonged breast feeding.

The present study had several limitations. Regarding the method used to measure iron concentration, serum ferritin is an acute-phase reactant that may be artificially elevated in the presence of inflammation [43], a potential confounding factor that we did not rule out (e.g., by adjusting for C-reactive protein, which was not measured). We did, however, exclude infants with acute or chronic inflammation or infection. Second, we did not measure urinary cotinine levels in these infants and did not exclude cadmium exposure due to passive smoking. Third, the lack of statistical significance might be due to the small sample size of the present study population. In particular, 12 iron-deficient infants were lost to follow up. The GM blood cadmium concentration in the remaining 19 infants was mildly decreased by ferric hydroxide treatment, but the difference was not statistically significant. If there had been no infants lost to follow-up, this difference may have reached statistical significance. Further studies with larger sample sizes are needed.

## Conclusion

In conclusion, we have shown here that iron deficiency does not increase blood cadmium concentrations in infants.

## Competing interests

The authors declare that they have no competing interests.

## Authors' contribution

YK, SP, and J-HP contributed to the conception and design of the study. YK is involved in the statistical analysis and interpretation of data. J-HP drafted the manuscript. All authors read and approved the final manuscript.
